# The first transcriptomes from field-collected individual whiteflies ( *Bemisia tabaci*, Hemiptera: Aleyrodidae):  a case study of the endosymbiont composition

**DOI:** 10.12688/gatesopenres.12783.3

**Published:** 2018-03-08

**Authors:** Peter Sseruwagi, James Wainaina, Joseph Ndunguru, Robooni Tumuhimbise, Fred Tairo, Jian-Yang Guo, Alice Vrielink, Amanda Blythe, Tonny Kinene, Bruno De Marchi, Monica A. Kehoe, Sandra Tanz, Laura M. Boykin

**Affiliations:** 1 Mikocheni Agriculture Research Institute (MARI), Dar es Salaam, P.O. Box 6226, Tanzania; 2 School of Molecular Sciences and Australian Research Council Centre of Excellence in Plant Energy Biology, University of Western Australia, Perth, WA, 6009, Australia; 3 National Agricultural Research Laboratories, P.O. Box 7065, Kampala Kawanda - Senge Rd, Kampala, Uganda; 4 Ministry of Agriculture Key Laboratory of Agricultural Entomology, Institute of Insect Sciences, Zhejiang University, Hangzhou, 310058, China; 5 State Key Laboratory for the Biology of Plant Diseases and Insect Pests, Institute of Plant Protection, Chinese Academy of Agricultural Sciences, Beijing, 100193, China; 6 Faculdade de Ciências Agronômicas, Universidade Estadual Paulista , Botucatu, Brazil; 7 Department of Primary Industries and Regional Development, DPIRD Diagnostic Laboratory Services, South Perth, WA, Australia

**Keywords:** Bacterial endosymbionts, sub-Saharan Africa, cassava, smallholder farmers, NusG, next generation sequencing

## Abstract

**Background:** *Bemisia tabaci* species ( *B. tabaci*), or whiteflies, are the world’s most devastating insect pests. They cause billions of dollars (US) of damage each year, and are leaving farmers in the developing world food insecure. Currently, all publically available transcriptome data for *B. tabaci* are generated from pooled samples, which can lead to high heterozygosity and skewed representation of the genetic diversity. The ability to extract enough RNA from a single whitefly has remained elusive due to their small size and technological limitations.

**Methods: **In this study, we optimised a single whitefly RNA extraction procedure, and sequenced the transcriptome of four individual adult Sub-Saharan Africa 1 (SSA1) *B. tabaci.* Transcriptome sequencing resulted in 39-42 million raw reads. *De novo* assembly of trimmed reads yielded between 65,000-162,000 Contigs across *B. tabaci* transcriptomes.

**Results: **Bayesian phylogenetic analysis of mitochondrion cytochrome I oxidase (mtCOI) grouped the four whiteflies within the SSA1 clade. BLASTn searches on the four transcriptomes identified five endosymbionts; the primary endosymbiont *Portiera* *aleyrodidarum* and four secondary endosymbionts: *Arsenophonus, Wolbachia, Rickettsia, *and *Cardinium spp. *that were predominant across all four SSA1 B. *tabaci* samples with prevalence levels of between 54.1 to 75%. Amino acid alignments of the *Nus*G gene of *P. aleyrodidarum *for the SSA1 *B. tabaci* transcriptomes of samples WF2 and WF2b revealed an eleven amino acid residue deletion that was absent in samples WF1 and WF2a. Comparison of the protein structure of the *Nus*G protein from *P. aleyrodidarum* in SSA1 with known *Nus*G structures showed the deletion resulted in a shorter D loop.

**Conclusions: **The use of field-collected specimens means time and money will be saved in future studies using single whitefly transcriptomes in monitoring vector and viral interactions. Our method is applicable to any small organism where RNA quantity has limited transcriptome studies.

## Introduction

Members of the whitefly *Bemisia tabaci* (Hemiptera: Aleyrodidae) species complex are classified as the world’s most devastating insect pests. There are 34 species globally ^1^ and the various species in the complex are morphologically identical. They transmit over 100 plant viruses ^2, 3^, become insecticide resistant ^4^, and ultimately cause billions of dollars in damage annually for farmers ^5^. The adult whiteflies are promiscuous feeders, and will move between viral infected crops and native weeds that act as viral inoculum ‘sources’, and deposit viruses to alternative crops that act as viral ‘sinks’ while feeding.

The crop of importance for this study was cassava ( *Manihot esculenta*). Cassava supports approximately 800 million people in over 105 countries as a source of food and nutritional security, especially within rural smallholder farming communities ^6^. Cassava production in Sub-Saharan Africa (SSA), especially the East African region, is hampered by whitefly-transmitted DNA and RNA viruses.

Among the DNA viruses, nine cassava-infecting cassava mosaic begomoviruses (CMBs) including: *African cassava mosaic virus* (ACMV), *African cassava mosaic Bukina faso virus* (ACMBV), *African cassava mosaic Madagascar virus* (ACMMV), *East African cassava mosaic virus* (EACMV), *East African cassava mosaic Cameroon virus* (EACMCV), *East African cassava mosaic Kenya virus* (EACMKV), *East African cassava mosaic Malawi virus* (EACMMV), *East African cassava mosaic Zanzibar virus* (EACMZV) and *South African cassava mosaic virus* (SACMV) have been reported in SSA ^7^. The DNA viruses cause cassava mosaic disease (CMD) leading to 28–40% crop losses with estimated economic losses of up to $2.7 billion dollars per year in SSA ^8^. The CMD pandemics in East Africa, and across other cassava producing areas in SSA, were correlated with *B. tabaci* outbreaks ^9^ Relevant to this study are two RNA ipomoviruses, in the family *Potyviridae* : *Cassava brown streak virus* (CBSV) and the *Ugandan cassava brown streak virus* (UCBSV), both devastating cassava in East Africa. *Bemisia tabaci* species have been hypothesized to transmit these RNA viruses with limited transmission efficiency ^10, 11^. Recent studies have shown that there are multiple species of these viruses ^12^, which further strengthens the need to obtain data from individual whiteflies as pooled samples could contain different species with different virus composition and transmission efficiency. In addition, CBSV has been shown to have a higher rate of evolution than UCBSV ^13^ increasing the urgency of understanding the role played by the different whitefly species in the system.

### Endosymbionts and their role in *B. tabaci*

Viral-vector interactions within *B. tabaci* are further influenced by bacterial endosymbionts forming a tripartite interaction. *B. tabaci* has one of the highest numbers of endosymbiont bacterial infections with eight different vertically transmitted bacteria reported ^14–17^. They are classified into two categories; primary (P) and secondary (S) endosymbionts, many of which are in specialised cells called bacteriocytes, while a few are also found scattered throughout the whitefly body. A single obligate *P-endosymbiont P. aleyrodidarum* is systematically found in all *B. tabaci* individuals. *Portiera* has a long co-evolutionary history with all members of the *Aleyrodinae* subfamily ^16^. In this study, we further explore genes within the *P. aleyrodidarum* retrieved from individual whitefly transcriptomes, including the transcription termination/antitermination protein *Nus*G. *Nus*G is a highly conserved protein regulator that suppresses RNA polymerase, pausing and increasing the elongation rate ^18, 19^. However, its importance within gene regulation is species specific; in *Staphylococcus aureus* it is dispensable ^20, 21^.

The S-endosymbionts are not systematically associated with hosts, and their contribution is not essential to the survival and reproduction. Seven facultative S-endosymbionts, *Wolbachia, Cardinium, Rickettsia, Arsenophonus, Hamiltonella defensa* and *Fritschea bemisae* and *Orientia-like organism* have been detected in various *B. tabaci* populations ^14, 22– 25^. The presence of S-endosymbionts can influence key biological parameters of the host. *Hamiltonella* and *Rickettsia* facilitate plant virus transmission with increased acquisition and retention by whiteflies ^25^. This is done by protection and safe transit of virions in the haemolymph of insects through chaperonins ( *GroEL*) and protein complexes that aid in protein folding and repair mechanisms ^20^.

### Application of next generation sequencing in pest management of *B. tabaci*

The advent of next generation sequencing (NGS) and specifically transcriptome sequencing has allowed the unmasking of this tripartite relationship of vector-viral-microbiota within insects ^24, 26– 28^. Furthermore, NGS provides an opportunity to better understand the co-evolution of *B. tabaci* and its bacterial endosymbionts ^26^. The endosymbionts have been implicated in influencing species complex formation in *B. tabaci* through conducting sweeps on the mitochondrial genome ^27^. Applying transcriptome sequencing is essential to reveal the endosymbionts and their effects on the mitogenome of *B. tabaci,* and predict potential hot spots for changes that are endosymbiont induced.

Several studies have explored the interaction between whitefly and endosymbionts ^29, 30^ and have resulted in the identification of candidate genes that maintain the relationship ^31, 32^. This has been explored as a source of potential RNAi pesticide control targets ^32– 34^. RNAi-based pest control measures also provide opportunities to identify species-specific genes for target gene sequences for knock-down. However, to date all transcriptome sequencing has involved pooled samples, obtained through rearing several generations of isolines of a single species to ensure high quantities of RNA for subsequent sequencing. This remains a major bottle neck in particular within arthropoda, where collected samples are limited due to small morphological sizes ^32^. In addition, the development of isolines is time consuming and often has colonies dying off mainly due to inbreeding depression ^34^.

It is against this background that we sought to develop a method for single whitefly transcriptomes to understand the virus diversity within different whitefly species. We did not detect viral reads, probably an indication that the sampled whitefly was not carrying any viruses, but as proof of concept of the method, we validated the utility of the data generated by retrieving the microbiota *P-endosymbionts* and *S-endosymbionts* that have previously been characterised within *B. tabaci*. In this study we report the endosymbionts present within field-collected individual African whiteflies, as well as characterisation and evolution of the *Nus*G genes present within the *P-endosymbionts*.

## Methods

### Whitefly sample collection and study design

In this study, we sampled whiteflies in Uganda and Tanzania from cassava ( *Manihot esculenta*) fields. In Uganda, fresh adult whiteflies were collected from cassava fields at the National Crops Resources Research Institute (NaCRRI), Namulonge, Wakiso district, which is located in central Uganda at 32°36’E and 0°31’N, and 1134 meters above sea level. The whiteflies obtained from Tanzania were collected on cassava in a countrywide survey conducted in 2013. The samples: WF2 (Uganda) and WF1, WF2a, and WF2b (Tanzania) used in this study were collected on CBSD-symptomatic cassava plants. In all the cases, the whitefly samples were kept in 70% ethanol in Eppendorf tubes until laboratory analysis. The whitefly samples were used for a two-fold function; firstly, to optimise a single whitefly RNA extraction protocol and secondly, to unmask RNA viruses and endosymbionts within *B. tabaci* as a proof of concept. In addition, we obtained a *Nus*G sequence from a Brazilian NW2 isolate (De Marchi, unpublished) and other downloaded and published *Nus*G sequences from GeneBank) to ensure phylogenetic representation across whitely species.

### Extraction of total RNA from single whitefly

RNA extraction was carried out using the ARCTURUS ^®^ PicoPure ^®^ kit (Arcturus, CA, USA), which is designed for fixed paraffin-embedded (FFPE) tissue samples. Briefly, 30 µl of extraction buffer were added to an RNase-free micro centrifuge tube containing a single whitefly and ground using a sterile plastic pestle. To the cell extract an equal volume of 70% ethanol was added. To bind the RNA onto the column, the RNA purification columns were spun for two minutes at 100 x g and immediately followed by centrifugation at 16,000 x g for 30 seconds. The purification columns were then subjected to two washing steps using wash buffer 1 and 2 (ethyl alcohol). The purification column was transferred to a fresh RNase-free 0.5 ml micro centrifuge tube, with 30 µl of RNAse-free water added to elute the RNA. The column was incubated at room temperature for five minutes, and subsequently spun for one minute at 1,000 x g, followed by 16,000 x g for one minute. The eluted RNA was returned into the column and re-extracted to increase the concentration. Extracted RNA was treated with DNase using the TURBO DNA free kit, as described by the manufacturer (Ambion, Life Technologies, CA, USA). Concentration of RNA was done in a vacuum centrifuge (Eppendorf, Germany) at room temperature for 1 hour, the pellet was suspended in 15 µl of RNase-free water and stored at -80°C awaiting analysis. RNA was quantified, and the quality and integrity assessed using the 2100 Bioanalyzer (Agilent Technologies, CA, USA). Dilutions of up to x10 were made for each sample prior to analysis in the bioanalyzer.

### cDNA and Illumina library preparation

Total RNA from each individual whitefly sample was used for cDNA library preparation using the Illumina TruSeq Stranded Total RNA Preparation kit as described by the manufacturer (Illumina, CA, USA). Subsequently, sequencing was carried out using the HiSeq2000 (Illumina) on the rapid run mode generating 2 x 50 bp paired-end reads. Base calling, quality assessment and image analysis were conducted using the HiSeq control software v1.4.8 and Real Time Analysis v1.18.61 at the Australian Genome Research Facility (Perth, Australia).

### Analysis of NGS data using the supercomputer

**Assembly of RNA transcripts:** Raw RNA-Seq reads were trimmed using Trimmomatic. The trimmed reads were used for *de novo* assembly using Trinity ^35^ with the following parameters: time -p srun --export=all -n 1 -c ${NUM_THREADS} Trinity --seqType fq --max_memory 30G --left 2_1.fastq --right 2_2.fastq --SS_lib_type RF --CPU ${NUM_THREADS} --trimmomatic --cleanup --min_contig_length 1000 -output _trinity min_glue = 1, V = 10, edge-thr = 0.05, min_kmer_cov = 2, path_reinforcement_distance = 150, and group pairs distance = 500.

**BLAST analysis of transcripts and annotation:** BLAST searches of the transcripts under study were carried out on the NCBI non-redundant nucleotide database using the default cut-off on the Magnus Supercomputer at the Pawsey Supercomputer Centre Western Australia. Transcripts identical to known bacterial endosymbionts were identified and the number of genes from each identified endosymbiont bacteria determined.

**Phylogenetic analysis of whitefly mitochondrial cytochrome oxidase I (COI):** The phylogenetic relationship of mitochondrial cytochrome oxidase I (mtCOI) of the whitefly samples in this study were inferred using a Bayesian phylogenetic method implemented in MrBayes (version 3.2.2) ^36^. The optimal substitution model was selected using Akaike Information Criteria (AIC) implemented in the Jmodel test ^37^.

**Sequence alignment and phylogenetic analysis of *Nus*G gene in *P. aleyrodidarum* across *B. tabaci* species:** Sequence alignment of the *Nus*G gene from the P-endosymbiont *P. aleyrodidarum* from the SSA1 *B. tabaci* in this study was compared with another *B. tabaci* species, *Trialeurodes vaporariorum* and *Alerodicus dispersus* using MAFFT (version 7.017) ^38^. The Jmodel version 2 ^37^ was used to search for phylogenetic models with the Akaike information criterion selecting the optimal that was to be implemented in MrBayes 3.2.2. MrBayes run was carried out using the command: “lset nst=6 rates=gamma” for 50 million generations, with trees sampled every 1000 generations. In each of the runs, the first 25% (2,500) trees were discarded as burn in.

### Analysis and modelling the structure of the *Nus*G gene

The structures for *Portiera aleyrodidarum BT* and *B. tabaci* SSA1 whitefly were predicted using Phyre2 ^39^ with 100% confidence and compared to known structures of *Nus*G from other bacterial species. All models were prepared using Pymol (The PyMOL Molecular Graphics System, Version 1.5.0.4).

## Results

### RNA extraction and NGS optimised for individual *B. tabaci* samples

In this study, we sampled four individual adult *B. tabaci* from cassava fields in Uganda (WF2) and Tanzania (WF1, WF2a, WF2b). Total RNA from single whitefly yielded high quality RNA with concentrations ranging from 69 ng to 244 ng that were used for library preparation and subsequent sequencing with Illumina Hiseq 2000 on a rapid run mode. The number of raw reads generated from each single whitefly ranged between 39,343,141 and 42,928,131 ( Table 1). After trimming, the reads were assembled using Trinity resulting in 65,550 to 162,487 transcripts across the four SSA1 *B. tabaci* individuals ( Table 1).

**Table 1.** Summary statistics from De novo Trinity assemble of Illumina paired end individual whitefly transcriptomes.

	WF1	WF2	WF2a	WF2b
Total Number of reads	39,343,141	42,587,057	42,513,188	42,928,131
Number of reads after trimming for quality	34,470,311 (87.61%)	39,898,821 (93.69%)	40,121,377 (94.37%)	40,781,932 (95.00%)
Transcripts	65,550	73,107	162,487	104,539
Number of endosymbiont contigs matching core genes	417	446	568	569
All transcript Contigs (N50)	505	525	1,084	1,018
Only longest Contigs (N50)	468	484	707	746

### Comparison of endosymbionts within the SSA1 *B. tabaci* samples

Comparison of the diversity of bacterial endosymbionts across individual whitefly transcripts was conducted with BLASTn searches on the non-redundant nucleotide database and by identifying the number of genes from each bacterial endosymbiont ( Supplementary Table 1). We identified five main endosymbionts including: *P. aleyrodidarum*, the primary endosymbionts and four secondary endosymbionts: *Arsenophonus, Wolbachia, Rickettsia sp, and Cardinium* spp ( Table 2). *P. aleyrodidarum* predominate across all four SSA1 *B. tabaci* study samples based on number of core gene families identified for WF1 (74.82%), WF2 (72.18%), WF2a (53.17%) and WF2b (71.70%). This was followed by *Arsenophonus*, *Wolbachia, Rickettsia sp,* and *Cardinium* spp, which occurred at an average of 18.0%, 5.9%, 1.6% and <1%, respectively across all four study samples ( Table 2). The secondary endosymbiont *Hamiltonella* had the least number of core genes (n=1) and was detected in only one of the SSA1 *B. tabaci* (WF2a) samples.

**Table 2.** Number of Contigs matching the core genes of bacteria endosymbionts across the four SSA1 whitefly transcriptome.

		Number of Contigs matching core genes of respective endosymbionts using BLAST			
	Endosymbionts	WF1	WF2	WF2a	WF2b
**Primary**	*Portiera*	312 (74.82%)	322 (72.18%)	302 (53.17%)	408 (71.70%)
**Secondary**	*Hamiltonella*	NA	NA	1 (0.002%)	NA
	*Rickettsia*	11 (2.64%)	8 (1.79%)	147 (25.88%)	NA
	*Wolbachia*	32 (7.67%)	25	71 (12.5%)	46 (8.08%)
	*Cardinium*	NA	NA	9 (1.58%)	6 (1.05%)
	*Fritschea*	NA	NA	NA	NA
	*Arsenophonus*	62 (14.87%)	91 (20.40%)	38 (6.69%)	109 (19.16%)
	**Total**	417	446	568	569

### Phylogenetic analysis of single whitefly mitochondrial cytochrome oxidase I (COI)

*B. tabaci* is recognized as a species complex of 34 species based on the mitochondrion cytochrome oxidase I ^1, 38, 39^. We therefore used cytochrome oxidase I (COI) transcripts of the four individual whitefly to ascertain *B. tabaci* species status and their phylogenetic relation using reference *B. tabaci* COI GenBank sequences found at http://www.whiteflybase.org. All four COI sequences clustered within Sub-Saharan Africa 1 clade (SSA1) species with greater than 99% identity ( www.whiteflybase.org).

### Sequence alignment and Bayesian phylogenetic analysis of *Nus*G gene

**Figure 1.**
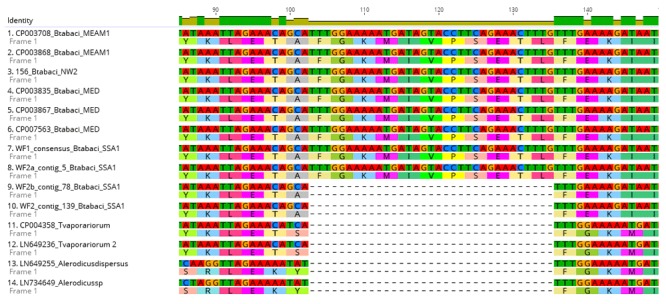
Sequence alignment of nucleotide sequences of *Nus*G gene in *P. aleyrodidarum* across whitefly species sequences using MAFFT v 7.017.

**Figure 2.**
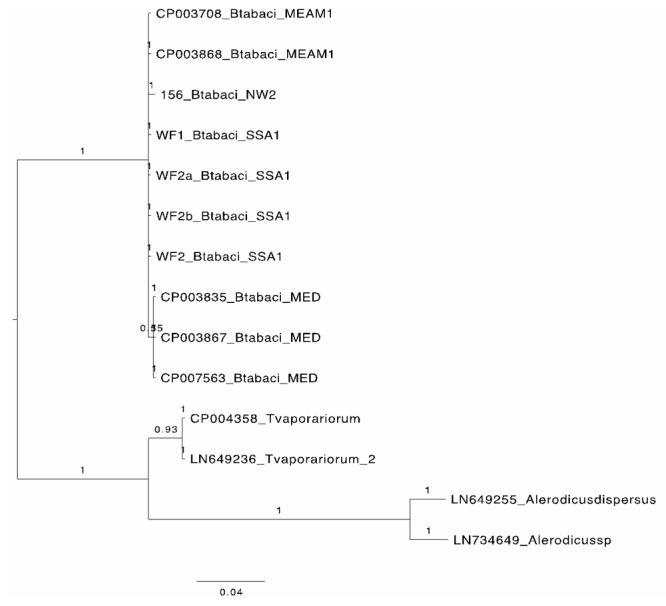
Bayesian phylogenetic tree of *Nus*G gene of *P. aleyrodidarum* across whitefly species using MrBayes -3.2.2.

Nucleotide and amino acid sequence alignments of the *Nus*G in *P. aleyrodidarum* were conducted for several whitefly species including: *B. tabaci* (SSA1), Mediterranean (MED) and Middle East Asia Minor 1 (MEAM1), New World 2 (NW2), *T. vaporariorum* (Greenhouse whitefly) and *Aleurodicus dispersus*. The alignment identified 11 missing amino acids in the *Nus*G sequences for the SSA1 *B. tabaci* samples: WF2 and WF2b, *T. vaporariorum* (Greenhouse whitefly) and *Aleurodicus dispersus.* However, all 11 amino acids were present in samples WF1 and WF2a, MED, MEAM1 and NW2 ( Figure 1). Bayesian phylogenetic relationships of the *Nus*G sequences of *P. aleyrodidarum* for the different whitefly species clustered all four SSA1 *B. tabaci* (WF1, WF2, WF2a and WF2b) within a single clade together with ancestral *B. tabaci* from GenBank ( Figure 2). The SSA1 clade was supported by posterior probabilities of 1 with *T. vaporariorum* and *Aleurodicus*, which formed clades at the base of the phylogenetic tree ( Figure 2).

### Structure analysis of Portiera *Nus*G genes

**Figure 3.**
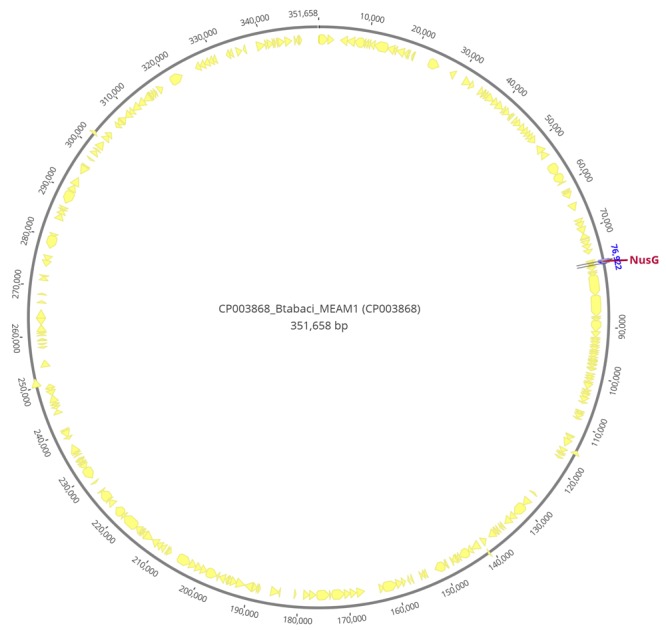
Primary endosymbiont *Portiera aleyrodidarum* whole genome from GenBank CP003868 showing the section of the NusG gene included in the analyses (position 76,922).

**Figure 4.**
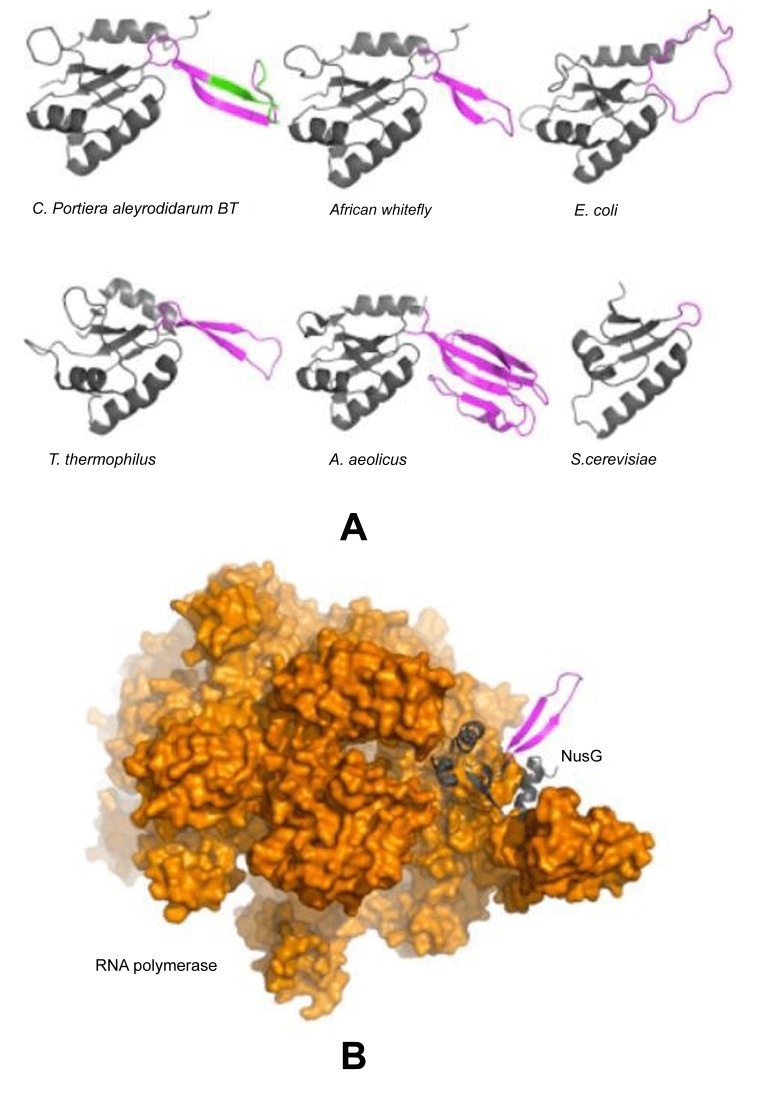
Structure analysis of *Nus*G from *P. aleyrodidarum* in *B. tabaci* and other endosymbionts. **A**. Phyre2 based structure prediction of *Nus*G from *Candidatus Portiera aleyrodidarum* in *B. tabaci* SSAI whitefly and comparisons to the structures of *Nus*G from other bacterial species as indicated and of Spt4/5 from yeast. *Nus*G is coloured in grey, the loop region in magenta and the 11-residue deletion is shown in green in the C. *Portiera aleyrodidarum* structure. **B**. A model of bacterial RNA polymerase (orange surface representation) bound to the N-terminal domain of the *T. thermophiles Nus*G (grey cartoon representation).

Structures of the *Nus*G protein sequence of the primary endosymbiont *P. aleyrodidarum* in the four SSA1 *B. tabaci* samples were predicated using Phyre2 with 100% confidence, and compared to known structures of *Nus*G from other bacterial species including ( *Shigella flexneri*, *Thermus thermophilus* and *Aquifex aeolicus*; (PDB entries 2KO6, 1NZ8 and 1M1H, respectively) and Spt4/5 from yeast ( *Saccharomyces cerevisiae;* PDB entry 2EXU) ^18, 40, 41^. The 11-residue deletion was found in a loop region that is variable in length and structure across bacterial species, but is absent from archaeal and eukaryotic species ( Figure 3 and Figure 4A). The effect of the deletion appears to shorten the loop in *Nus*G from the African whiteflies (WF2 and WF2b). A model of bacterial RNA polymerase (orange surface representation; PDB entry 2O5I) bound to the N-terminal domain of the *Thermus thermophilus Nus*G shows that the loop region is not involved in the interaction between *Nus*G and RNA polymerase ( Figure 4B).

## Discussion

In this study, we optimised a single whitefly RNA extraction method for field-collected samples. We subsequently successfully conducted RNA sequencing on individual Sub-Saharan Africa 1 (SSA1) *B. tabaci*, revealing unique genetic diversity in the bacterial endosymbionts as proof of concept. This is the first time a single whitefly transcriptome has been produced.

### *Nus*G deletion and implications within *P. aleyrodidarum* in SSA *B. tabaci*

We report the presence of the primary endosymbionts *P. aleyrodidarum* and several secondary endosymbionts within SSA1 transcriptome. Furthermore, *P. aleyrodidarum* in SSA1 *B. tabaci* was observed to have a deletion of 11 amino acids on the *Nus*G gene that is associated with cellular transcriptional processes within another bacteria species. On the other hand, *P. aleyrodidarum* from NW2, MED and SSA1 (WF2a, WF1) *B. tabaci* species did not have this deletion ( Figure 1). The deleted 11 amino acids were identified in a loop region of the N-terminal domain of *Nus*G protein, resulting in a shortened loop in the SSA1 WF2b sample. This loop region has high variability in both structure and length across bacterial species, and is absent from archaea and eukaryotic species.

*Nus*G is highly conserved and a major regulator of transcription elongation. It has been shown to directly interact with RNA polymerase to regulate transcriptional pausing and rho-dependent termination ^15, 20, 42, 43^. Structural modelling of *Nus*G bound to RNA polymerase indicated that the shortened loop region seen in the WF2b sample is unlikely to affect this interaction. Rho-dependant termination has been attributed to the C-terminal (KOW) domain region of *Nus*G, therefore a shortening of the loop region in the N-terminal domain is also unlikely to affect transcription termination. Yet, there has been no function attributed to this loop region of *Nus*G, and thus the effect of variability in this region across species is unknown. However, the deletion could represent the results of evolutionary species divergence. Further sequencing of the gene is required across the *B. tabaci* species complex to gain further understanding of the diversity.

### Why the single whitefly transcriptome approach?

The sequencing of the whitefly transcriptome is crucial in understanding whitefly-microbiota-viral dynamics and thus circumventing the bottlenecks posed in sequencing the whitefly genome. The genome of whitefly is highly heterozygous ^42^. Assembling of heterozygous genomes is complex due to the de Bruijn graph structures predominantly used ^43^. To deal with the heterozygosity, previous studies have employed inbred lines, obtained from rearing a high number of whitefly isolines ^35, 44^. However, rearing whitefly isolines is time consuming and often colonies may suffer contaminations, leading to collapse and failure to raise the high numbers required for transcriptome sequencing.

We optimised the ARCTURUS ^®^ PicoPure ^®^ kit (Arcturus, CA, USA) protocol for individual whitefly RNA extraction with the dual aim of determining if we could obtain sufficient quantities of RNA from a single whitefly for transcriptome analysis and secondly, determine whether the optimised method would reveal whitefly microbiota as proof of concept. Using our method, the quantities of RNA obtained from field-collected single whitefly samples were sufficient for library preparation and subsequent transcriptome sequencing. Across all transcriptomes over 30M reads were obtained. The amount of transcripts were comparable to those reported in other arthropoda studies from field collections ^32^. However, we did not observe any difference in assembly qualities ^32^; probably due to the fact that our field-collected samples had degraded RNA based on RIN, and thus direct comparison with 32 was inappropriate.

Degraded insect specimen have been used successfully in previous studies ^45^. This is significant, considering that the majority of insect specimens are usually collected under field conditions and stored in ethanol with different concentrations ranging from 70 to 100% ^46– 48^ rendering the samples liable to degradation. However, to ensure good keeping of insect specimen to be used for mRNA and total RNA isolation in molecular studies, and other downstream applications such as histology and immunocytochemistry, it is advisable to collect the samples in an RNA stabilizing solution such as RNAlater. The solution stabilizes and protects cellular RNA in intact, unfrozen tissue, and cell samples without jeopardizing the quality, or quantity of RNA obtained after subsequent RNA isolation. The success of the method provided an opportunity to unmask vector-microbiota-viral dynamics in individual whiteflies in our study, and will be useful for similar studies on other small organisms.

### Endosymbionts diversity across individual SSA1 *B. tabaci* transcriptomes

In this study, we identified bacterial endosymbionts ( Table 2) that were comparable to those previously reported in *B. tabaci* ^49^ and more specifically SSA1 on cassava ^23, 37^. Secondary endosymbionts have been implicated with different roles within *B. tabaci*. *Rickettsia* has been adversely reported across putative *B. tabaci* species, including the Eastern African region ^23, 50, 51^. This endosymbiont has been associated with influencing thermo tolerance in *B. tabaci* species ^49^. *Rickettsia* has also been associated with altering the reproductive system of *B. tabaci,* and within the females. This has been attributed to increasing fecundity, greater survival, host reproduction manipulation and the production of a higher proportion of daughters all of which increase the impact of virus ^49^. In addition, *Rickettsia* and *Hamiltonella* play a role in plant virus transmission in whiteflies ^25^ by protecting the safe transit of virions in the haemolymph of insects through chaperonins ( *GroEL*) and protein complexes that aid in protein folding and repair mechanisms ^20^. However, *Hamiltonella* was reported to be absent in the indigenous whitefly populations studied elsewhere ^15, 50, 52– 54^ and in Malawi, Nigeria, Tanzania and Uganda ^50, 55^ as also confirmed in our study. *Arsenophonus, Wolbachia, Arsenophonus* and *Cardinium spp* have been detected within MED and MEAM1 *Bemisia* species ^14, 50^. In addition, 50 and 22 reported *Arsenophonus* within SSA1 *B. tabaci* in Eastern Africa that were collected on cassava. These endosymbionts have been associated with several deleterious functions within *B. tabaci* that include manipulating female-male host ratio through feminizing genetic males, coupled with male killing ^56, 57^.

Within the context of SSA agricultural systems, the role of endosymbionts in influencing *B. tabaci* viral transmission is important. Losses attributed to *B. tabaci* transmitted viruses within different crops are estimated to be in billions of US dollars ^46^. Bacterial endosymbionts have been associated with influencing viral acquisition, transmission and retention, such as in tomato leaf curl virus ^58, 59^. Thus, better understanding of the diversity of the endosymbionts provides additional evidence on which members of *B. tabaci* species complex more proficiently transmit viruses, and thus the need for concerted efforts towards the whitefly eradication.

## Conclusions

Our study provides a proof of concept that single whitefly RNA extraction and RNA sequencing is possible and the method could be optimised and applicable to a range of small insect transcriptome studies. It is particularly useful in studies that wish to explore vector-microbiota-viral dynamics at individual insect level rather than pooling of insects. It is useful where genetic material is both limited, as well as of low quality, which is applicable to most agriculture field collections. In addition, the single whitefly RNA sequencing technique described in this study offers new opportunities to understand the biology, and relative economic importance of the several whitefly species occurring in ecosystems within which food is produced in Sub-Saharan Africa, and will enable the efficient development and deployment of sustainable pest and disease management strategies to ensure food security in the developing countries. However, this method still requires further optimisation to recover viral reads, especially in cases with very low viral titre as observed in this study. Finally future studies could use freshly collected whiteflies on CBSD-affected plants to increase the detection of the causal viruses.

## Data availability

The datasets used and/or analyzed during the current study are available from GenBank:

SRR5110306, SRR5110307, SRR5109958, KY548924, MG680297.

## Supplementary material

**Supplementary Table 1.** Distribution of the endosymbionts and number of contigs matching core genes present within each endosymbiont bacteria present in four SSA1 *B. tabaci* samples from this study.
