# Supplementary material for: The first transcriptomes from field-collected individual whiteflies ( Bemisia tabaci, Hemiptera: Aleyrodidae): a case study of the endosymbiont composition
Source: Gates Open Res. 2018 Mar 8;1:16. Originally published 2017 Dec 28. [Version 3] doi: 10.12688/gatesopenres.12783.3 (PMC5872585; doi:10.12688/gatesopenres.12783.3)
Supplement: Supplementary file 1 [file gatesopenres-1-13874-s0000.tgz › e7acdc20-56b2-4590-9e16-a283b63c069e.doc]

**Supplementary Table 1.** Distribution of the endosymbionts and number of contigs matching core genes present within each endosymbiont bacteria present in four SSA1 *B. tabaci* samples from this study.

| **Endosymbionts** | **WF2** | **WF2a** | **WF2b** | **WF1** |
| --- | --- | --- | --- | --- |
| Candidatus Portiera aleyrodidarum | 322 | 302 | 408 | 312 |
| Arsenophonus | 3 | 1 | 1 | 1 |
| Arsenophonus endosymbiont of Bemisia tabaci | 1 | 2 | 1 | 2 |
| Arsenophonus endosymbiont of Nilaparvata lugens | 55 | 22 | 73 | 47 |
| Arsenophonus nasoniae | 33 | 13 | 34 | 12 |
| Wolbachia endosymbiont of Cadra cautella | 3 | 6 | 6 | 3 |
| Wolbachia endosymbiont of Caudra cautella | NA | NA | NA | 3 |
| Wolbachia endosymbiont of Cimex lectularius | NA | 5 | 2 | NA |
| Wolbachia endosymbiont of Culex quinquefasciatus | 2 | 9 | 3 | 2 |
| Wolbachia endosymbiont of Diaphorina citri | 3 | NA | 1 | 4 |
| Wolbachia endosymbiont of Drosophila ananassae | 6 | 14 | 15 | 6 |
| Wolbachia endosymbiont of Drosophila simulans | 1 | 6 | 5 | 3 |
| Wolbachia endosymbiont of Operophtera brumata | NA | 2 | NA | 2 |
| Wolbachia endosymbiont of Muscidifurax uniraptor | 1 | NA | NA | NA |
| Wolbachia endosymbiont wVitA of Nasonia vitripennis phage WOVitA1/Wolbachia endosymbiont wVitB of Nasonia vitripennis phage WOVitB | 1 | 6 | 3 | NA |
| Wolbachia pipientis | 5 | 9 | 4 | 4 |
| Wolbachia pipientis wAlbB | 5 | 4 | 2 | NA |
| Wolbachia sp. wRi | 3 | 13 | 8 | 5 |
| Rickettsia africae | NA | 2 | 1 | NA |
| Rickettsia argasii T170-B | NA | 4 | 7 | NA |
| Rickettsia australis | NA | 1 | 2 | NA |
| Rickettsia buchneri | NA | NA | 10 | NA |
| Rickettsia Canadensis | NA | 5 | NA | NA |
| Rickettsia Helvetica | 3 | 2 | 3 | 5 |
| Rickettsia hoogstraalii | NA | NA | 2 | NA |
| Rickettsia japonica | NA | 1 | NA | NA |
| Rickettsia massiliae MTU5 | NA | 1 | 3 | NA |
| Rickettsia monacensis | NA | NA | 5 | NA |
| Rickettsia prowazekii | NA | NA | 4 | NA |
| Rickettsia tamurae | NA | NA | 1 | NA |
| Rickettsia endosymbiont of Ixodes scapularis | 1 | 20 | 9 | 1 |
| Candidatus Rickettsia asemboensis | NA | NA | NA | 1 |
| Candidatus Rickettsia gravesii | NA | 1 | 1 | NA |
| Candidatus Rickettsia amblyommii str. Ac/Pa | NA | 1 | NA | NA |
| Candidatus Rickettsia amblyommii | NA | 1 | NA | NA |
| Candidatus Rickettsia amblyommii str. GAT-30V | NA | 1 | NA | NA |
| Rickettsiaceae bacterium Os18 | NA | 43 | 34 | 2 |
| Rickettsiales bacterium Ac37b | 1 | 4 | 4 | NA |
| Rickettsia peacockii str. Rustic | NA | 26 | 13 | NA |
| Rickettsia bellii | 1 | 10 | 10 | 1 |
| Rickettsia felis str. Pedreira | NA | 4 | 4 | 1 |
| Rickettsia felis str. LSU | NA | 5 | 8 | NA |
| Rickettsia prowazekii str. GvF12 | 2 | 2 | 4 | NA |
| Cardinium endosymbiont of Bemisia tabaci | NA | 4 | 3 | NA |
| Cardinium endosymbiont of Encarsia pergandiella | NA | 5 | 3 | NA |
| Candidatus Hamiltonella defensa | NA | 1 | NA | NA |
