# Supplementary material for: The first transcriptomes from field-collected individual whiteflies ( Bemisia tabaci, Hemiptera: Aleyrodidae):  a case study of the endosymbiont composition
Source: Gates Open Res. 2018 Mar 8;1:16. Originally published 2017 Dec 28. [Version 3] doi: 10.12688/gatesopenres.12783.3 (PMC5872585; doi:10.12688/gatesopenres.12783.3)
Supplement: Peer review history

# Peer review: Referee response for version 3

**DOI:** 10.21956/gatesopenres.13874.r26304

**Published:** 2018-03-15

**Authors:** Henryk H. Czosnek

**Affiliations:** 1 The Robert H. Smith Institute of Plant Sciences and Genetics in Agriculture, The Hebrew University of Jerusalem, Jerusalem, Israel

I am satisfied with the latest version, especially with the amended title, which reflects much better the content than before.

 I think this is a very good paper (it was also before – notwithstanding my comments), summarizing a very good piece of work and a true advance on the way to better understand the biology of whiteflies.

I have read this submission. I believe that I have an appropriate level of expertise to confirm that it is of an acceptable scientific standard.

# Peer review: Referee response for version 2

**DOI:** 10.21956/gatesopenres.13862.r26243

**Published:** 2018-02-26

**Authors:** Kai-Shu Ling

**Affiliations:** 1 USDA-ARS (United States Department of Agriculture - Agricultural Research Service), Charleston, SC, USA

The presentation in this revised version has been improved. The technology in generating relatively high numbers of sequence reads through RNA sequencing from field-collected individual whiteflies is quite impressive, which may have a broader implication for other researchers who are working in the similar situation with limited amount of material from a small insect or other organism for transcriptome analysis. 

 As a proof of concept, authors focused on their sequence analysis primarily on sequences derived from endosymbionts. Besides the mtCOI gene, typical transcriptome analysis, including transcript expression and gene ontology relating to the whitefly genome, was not conducted in the current study. Therefore, although authors prefer to maintain the original title, I am still have a reservation on the title as it does not reflect well the main content of the current presentation.

I have read this submission. I believe that I have an appropriate level of expertise to confirm that it is of an acceptable scientific standard.

# Response

**Published:** 2018-03-06

**Authors:** Laura Boykin

Thank you very much for your review. We have changed the title as you have suggested.

# Peer review: Referee response for version 2

**DOI:** 10.21956/gatesopenres.13862.r26244

**Published:** 2018-02-21

**Authors:** Henryk H. Czosnek

**Affiliations:** 1 The Robert H. Smith Institute of Plant Sciences and Genetics in Agriculture, The Hebrew University of Jerusalem, Jerusalem, Israel

I am still very impressed by the technical prowess consisting in developing a method that allows the analysis of the transcriptome of individual whiteflies. The manuscript has been improved by modifying some of the tables.

 Again, and the authors have not followed me in this, I believe they should go beyond the analysis of endosymbiont transcripts; they have all the data to do so. This way, they could present the advantages and the limits of single insect transcriptome analyses in deciphering the many aspects of whitefly biology. Limiting the analysis to symbionts in this paper is a waste of analytical power offered by the technology. In addition, I stated in my review that the title is deceiving, since the focus is on the endosymbiont-related transcriptome. Again, the authors preferred to retain their title. I could suggest the following: “The first transcriptomes from field-collected individual whiteflies allow to identify specific endosymbiont gene expression”. I believe this is critical for the “business card” of the paper.

I have read this submission. I believe that I have an appropriate level of expertise to confirm that it is of an acceptable scientific standard, however I have significant reservations, as outlined above.

# Response

**Published:** 2018-03-06

**Authors:** Laura Boykin

Thank you for your positive comments on our manuscript. We have changed the title of the paper to “ **The first transcriptomes from field-collected individual whiteflies ( *Bemisia tabaci*, Hemiptera: Aleyrodidae): a case study of the endosymbiont composition.**”. All data is available for other whitefly researchers to do the analyses suggested above.  The main focus of our paper was to publish the laboratory method. We are pleased the second reviewer agrees.

# Peer review: Referee response for version 1

**DOI:** 10.21956/gatesopenres.13843.r26202

**Published:** 2018-01-22

**Authors:** Henryk H. Czosnek

**Affiliations:** 1 The Robert H. Smith Institute of Plant Sciences and Genetics in Agriculture, The Hebrew University of Jerusalem, Jerusalem, Israel

In this paper, Sseruwagi *et al*. present a method of RNA preparation, which is suitable for the Illumina-based RNASeq analysis of the transcriptome of a single whitefly.

 Using this method, the authors have sequenced the transcriptome of four individuals (one from Uganda and three from Tanzania) collected on cassava leaves with symptoms said to be produced *by Cassava brown streak virus* (CBSV), a (+)ssRNA virus belonging to the *Potyviridae* family, probably transmitted by *B. tabaci* (still questioned in the literature).

 Indeed, this is an important technical feat. No doubt, it may help follow the movement of whiteflies and the diseases they transmit. In this context, it is interesting to note that the sequence and the functional analysis of the transcriptome of single cells has been published in several instances (recently reviewed by Liu and Trapnell, 2016).

 This paper is a bit disappointing, to me. The title claims that the authors have produced the first transcriptome of a single whitefly. This is true on its face value; and the results have been posted in GenBank (although, raw data). It should be relatively easy to identify transcripts since the sequence of *B. tabaci* MEAM1 and MED are known. Nonetheless, this reviewer is expecting some valuable information on gene expression in the whole animal. Is the lack of data in the paper due to a low number of reads of transcripts of cellular genes? Could the author identify, say, transcripts of housekeeping genes or genes involved in sugar metabolism or else? This would underline the power and the limits of the one-insect-one-transcriptome analysis.

 Instead, the authors have chosen to focus on the whitefly primary (P) and secondary (S) endosymbionts, especially on the NusG gene of the primary endosymbiont *Portiera aleyrodidarum* (4 figures). This gene might be interesting but it is more a structural than a functional study, which to my point of view lessens the importance of the study.

 The supplementary Table 1 is interesting but does not tell us the endosymbiont composition of the four individuals scrutinized. Is it Portiera (P), and Arsephonus, Wolbachia and Rickettsia? Also, the title of Table 1 is not clear; what is the meaning of “number of genes in endosymbionts bacteria”? Is it the number of genes with homologies to others?

 It is interesting that Hamiltonella sequences have not been found, knowing that this is the symbiont that produces GroEL, which binds to the CP of begomoviruses, and facilitates the transit of the virus in the hemolymph. It is also interesting that CBSV sequences were not found, although the whiteflies have been collected on symptomatic plants. Is it that, after all, *B. tabaci* is not the vector of this virus?

 Altogether, I expected much more from the title. I suggest to lower expectations of the reader by amending the title to something like “Analysis of the endosymbiont transcriptome from individual whiteflies”.

**I recommend publication after relating to the points mentioned above.**

I have read this submission. I believe that I have an appropriate level of expertise to confirm that it is of an acceptable scientific standard, however I have significant reservations, as outlined above.

# Response

**Published:** 2018-02-02

**Authors:** Laura Boykin

Your comments have greatly improved our manuscript and an updated version is now available for review. Thank you.

**Reviewer 2**

**Comments**

In this paper, Sseruwagi *et al*. present a method of RNA preparation, which is suitable for the Illumina-based RNASeq analysis of the transcriptome of a single whitefly.

Using this method, the authors have sequenced the transcriptome of four individuals (one from Uganda and three from Tanzania) collected on cassava leaves with symptoms said to be produced *by Cassava brown streak virus* (CBSV), a (+)ssRNA virus belonging to the *Potyviridae* family, probably transmitted by *B. tabaci* (still questioned in the literature).

Indeed, this is an important technical feat. No doubt, it may help follow the movement of whiteflies and the diseases they transmit. In this context, it is interesting to note that the sequence and the functional analysis of the transcriptome of single cells has been published in several instances (recently reviewed by Liu and Trapnell, 2016).

This paper is a bit disappointing, to me. The title claims that the authors have produced the first transcriptome of a single whitefly. This is true on its face value; and the results have been posted in GenBank (although, raw data). It should be relatively easy to identify transcripts since the sequence of *B. tabaci* MEAM1 and MED are known. Nonetheless, this reviewer is expecting some valuable information on gene expression in the whole animal. Is the lack of data in the paper due to a low number of reads of transcripts of cellular genes? Could the author identify, say, transcripts of housekeeping genes or genes involved in sugar metabolism or else? This would underline the power and the limits of the one-insect-one-transcriptome analysis.

**Response:**

*The initial experiment was meant to determine if we could obtain sufficient RNA and conduct RNAseq analysis on individual field collected B. tabaci. Our primary aim was to unravel the microbiota within individual transcriptome. Conducting gene expression analysis is still a challenge with the current method, mainly due to variation in starting RNA concentration of the whiteflies. Secondly, we did not achieve adequate ribosomal depletion, which may have hindered successful gene expression analysis. However, based on this method (ongoing) we have indeed identified nuclear genes and single copy orthologs.*

**Comments**

Instead, the authors have chosen to focus on the whitefly primary (P) and secondary (S) endosymbionts, especially on the NusG gene of the primary endosymbiont *Portiera aleyrodidarum* (4 figures). This gene might be interesting but it is more a structural than a functional study, which to my point of view lessens the importance of the study.

**Response:**

*We focused on the NusG mainly due to the unique deletion observed on what should be highly conserved proteins that are reported to be crucial in bacterial replication. It highlights the unique features of the endosymbionts from SSA species of B. tabaci compared to other putative species of B. tabaci and further highlights the difference within this species within the species complex. These findings were possible because we studied individual whitefly transcriptomes, and may probably not have been discovered by transcriptomes generated from pooled isolines.*

**Comments**

The supplementary Table 1 is interesting but does not tell us the endosymbiont composition of the four individuals scrutinized. Is it Portiera (P), and Arsephonus, Wolbachia and Rickettsia? Also, the title of Table 1 is not clear; what is the meaning of “number of genes in endosymbionts bacteria”? Is it the number of genes with homologies to others?

**Response:**

*We have revised and clarified the legends and content of both Tables 1 and 2 and supplementary Table1.*

**Comments**

It is interesting that Hamiltonella sequences have not been found, knowing that this is the symbiont that produces GroEL, which binds to the CP of begomoviruses, and facilitates the transit of the virus in the hemolymph.

**Response:**

*Candidatus Hamiltonella defensa has been reported to be absent in whiteflies in Africa. Our study found very negligible numbers of contigs in only one of the whiteflies (WF2a) studied. However, the literature (lines 117 to 120 in this paper) indicates that Rickettsia spp. is also involved in virus transmission, and is among the predominant endosymbionts detected in our study. We have added the results of Hamiltonella and Rickettsia to clarify the reviewer’s concerns.*

**Comment**

It is also interesting that CBSV sequences were not found, although the whiteflies have been collected on symptomatic plants. Is it that, after all, *B. tabaci* is not the vector of this virus?

**Response:**

*RNA viruses such as CBSVs are picked up and kept for short periods in the whitefly stylet, unlike the DNA viruses that build-up and keep long in the midgut, and are more likely to be detected if present in the whitefly under study. It is also possible that the whitefly were not viruliferous considering that less than 10% of field collected whiteflies are viruliferous despite them feeding on infected.*

*Secondly, a recent publication (Ateka E, Alicai T, Ndunguru J, Tairo F, Sseruwagi P, Kiarie, S., et al. (2017) Unusual occurrence of a DAG motif in the Ipomovirus Cassava brown streak virus and implications for its vector transmission. PLoS ONE 12(11): e0187883 reported the presence of a DAG motif within CBSVs indicating they could be aphid-transmitted viruses rather than by whiteflies.*

**Comment**

Altogether, I expected much more from the title. I suggest to lower expectations of the reader by amending the title to something like “Analysis of the endosymbiont transcriptome from individual whiteflies”.

**Response:**

*We appreciate the reviewers comment regarding the title but we prefer the current title as our study it is the first transcriptome generated from field collected whiteflies- the analyses pipelines can be investigated and expanded upon with future studies.*

# Peer review: Referee response for version 1

**DOI:** 10.21956/gatesopenres.13843.r26170

**Published:** 2018-01-16

**Authors:** Kai-Shu Ling

**Affiliations:** 1 USDA-ARS (United States Department of Agriculture - Agricultural Research Service), Charleston, SC, USA

Sseruwagi and colleagues in this manuscript described a method to effectively generate a high throughput RNA-seq dataset using purified total RNA extracted from each individual field-collected adult whitefly, *Bemisia tabaci*, which generated 39-42 million raw reads per library using Illumina sequencing. Because the genome sequence of cassava whitefly *B. tabaci* SSA-1 is yet available, through *de novo* assembly of cleaned reads, high number of contigs (65,000-162,000) from each library were generated. Functional prediction to profile the generated transcripts of *B. tabaci* SSA1 were not performed.  However, sequences to the mitochondrion cytochrome I oxidase (mtCOI) gene were identified from each of the four RNA-seq libraries. Phylogenetic analysis of mtCOI confirmed its close relationship to the cassava whitefly *B. tabaci* SSA1 clade. In addition, these RNA-seq datasets also contained sequences relating to five endosymbiont bacteria. Although authors claimed to have transcriptomes for these endosymbionts, extensive analysis to functionally profile the identified RNA sequences of these endosymbionts was not conducted in the current study. Individual analysis through amino acid alignment of the identified Nus G gene sequences in the primary *Portiera aleydidarum* from four RNA-seq datasets revealed an eleven amino acid residue deletion in two of the four individual whitefly libraries. Although this finding is interesting, a validation test would be necessary to confirm the missing sequences in those individuals through Sanger sequencing of amplicons generated using Nus G specific primers on the original RNA preparations. It is also surprising that not a single sequence relating to cassava-infecting viruses although these whiteflies were supposedly collected from cassava plants infected with cassava brown streak virus which has a poly-A tail in its RNA genome. It would be an interest to test the original RNA preparations to determine which viruses may be in these individual whiteflies. 

**Specific comments and suggestions:**

 Title: As mentioned in the general comments, this is more like a method paper in doing RNA sequencing on little RNA extracted from individual whitefly, not an extensive transcriptome analysis. I would suggest to change the title to something like this: Effective RNA sequencing using little RNA extracted from field-collected individual whiteflies ( *Bemisia tabaci*) useful for transcriptome analysis.

 IN ABSTRACT:

- Page 1: change 65,000-162,000 transcripts to contigs.
- Page 1: the compound sentence starting with “BLASTn searches …” This compound sentence is too long and the meaning is not clear, need to rewrite.

 

 IN INTRODUCTION:

- Page 3: need to modify the sentence ending with “…is hampered by both DNA and RNA (transmitted) virus”, either deleting “transmitted” or change the sentence to “is hampered by whitefly-transmitted DNA and RNA viruses”.
- Page 3: The sentence starting as “Relevant to this study are two RNA Potyviruses: …” Potyviruses should be replaced with “ipomoviruses, in the family Potyviridae”: …
- Page 3: in the same paragraph as above, you may want to elaborate a little bit more on virus species and genetic diversity, such as which viruses have been proven to be transmitted and which have not be proven to be transmitted by the SSA1 whiteflies and their efficiency.
- Page 3: Should be consistent in using P-endosymbiont, rather than P-symbiont and in other locations in the same document using P-endosymbiont.
- Page 3: You stated “Seven facultative S-endosymbionts…,” but only six endosymbionts are actually listed, need to add the missing one.
- Page 3: In the sentence starting with “It is against this background that we…” Your goal was to study whitefly-virus interaction, it is rather surprising that not a single virus sequence read was detected in these RNA-seq datasets in field-collected whiteflies. Rather than speculating these individual whitefly did not carry the target viruses, why not doing some tests by RT-PCR to confirm the lack of target viruses in these RNA preparations?

 

 IN METHODS:

- Page 4: Since whiteflies were collected from CBSD-symptomatic cassava plants in Uganda and Tanzania, it might still be possible to conduct RT-PCR tests to determine the presence of viruses in the purified whitefly  RNA preparations.
- Page 4: The rationale in using the Brazilian whitefly sample? Also it appears to me that there were no analysis of sequences from this Brazilian dataset in the result section.

 

 IN RESULTS:

- Page 5: There were 65,550 to 162,487 contigs generated from individual RNA-seq datasets. How all these sequences could be assigned to? Any ideas on what proportion of the sequences belonging to whitefly *B. tabaci *genome, what proportion of the sequences to endosymbionts?
- Page 5: The citation to Table 2 seems to point to the listing of five endosymbionts, however the content in the Table 2 showing the origin of whiteflies collected.
- Page 5: What are these incidences (74.8%, 71.2%, 54.1% and 58.5%) mean? The meaning is not clear, a proportion of total endosymbiont sequences that assigned to *P. aleyrodidarum*?
- Page 5:  The (data not shown) is not a good idea, it would be good to present it as a supplementary file.

IN DISCUSSION:

- Page 8: As mentioned in the general comment, you developed a method to effectively conduct RNA-sequencing on little RNA from individual whitefly and sequence analysis on specific genes in endosymbionts and COI gene of whitefly, I wouldn’t over use the term transcriptome analysis.
- Page 8: The deletion of 11 amino acids on the *Nus *G gene, this could be resulted from sequence assembly from short reads, therefore a validation of the deletion sequence on these whiteflies by RT-PCR and Sanger sequencing would be necessary, as pointed out by the authors that field collected samples may have resulted in RNA degradation in RNA purification, cDNA library preparation and/or sequencing.
- Page 9: The sentence starts: “In addition, 51 and 21 reported…”, what do these numbers (51 and 21) mean here? If there are citations to references, you need to use author name followed by the reference number.
- Page 9: Tomato leaf curl virus should be changed to tomato yellow leaf curl virus. 

 

 IN CONCLUSION:

- Page 9: Suggest to change transcriptome sequencing to RNA sequencing.
- Page 9: In this study, you only worked with whiteflies, in the sentence you need to change “is” to “could be optimized and applicable to …”
- Page 9: When you mentioned it is useful to study vector-microbiota-viral dynamics, but why not a single viral sequence read was detected in these datasets? Although there are some advantage in using RNA sequencing of individual whitefly, you may also want to point out there are still some room for improvement. 

I have read this submission. I believe that I have an appropriate level of expertise to confirm that it is of an acceptable scientific standard, however I have significant reservations, as outlined above.

# Response

**Published:** 2018-02-02

**Authors:** Laura Boykin

Thank you for your comments, they have greatly improved our manuscript. A new version is ready for review and our responses to each comment are listed below.

**Reviewer 1**

**Specific comments and suggestions:**

Title: As mentioned in the general comments, this is more like a method paper in doing RNA sequencing on little RNA extracted from individual whitefly, not an extensive transcriptome analysis. I would suggest changing the title to something like this: Effective RNA sequencing using little RNA extracted from field-collected individual whiteflies ( *Bemisia tabaci*) useful for transcriptome analysis.

**Response:**

*We appreciate the reviewers comment regarding the title but we prefer the current title as our study it is the first transcriptome generated from field-collected whiteflies- the analyses pipelines can be investigated and expanded upon with future studies.*

IN ABSTRACT:

Page 1: change 65,000-162,000 transcripts to contigs.

**Response:**

*Done*

Page 1: the compound sentence starting with “BLASTn searches …” This compound sentence is too long and the meaning is not clear, need to rewrite.

**Response:**

*Done*

For research articles we ask that you structure your abstract under subtitles of background, methods, results and conclusions. For more information please see section 3 of our research article guidelines.

**Response:**

*Done*

IN INTRODUCTION:

Page 3: need to modify the sentence ending with “…is hampered by both DNA and RNA (transmitted) virus”, either deleting “transmitted” or change the sentence to “is hampered by whitefly-transmitted DNA and RNA viruses”.

**Response:**

*Corrected to read ….” is hampered by whitefly-transmitted DNA and RNA viruses”*

Page 3: The sentence starting as “Relevant to this study are two RNA Potyviruses: …” Potyviruses should be replaced with “ipomoviruses, in the family Potyviridae”: …

**Response:**

*Corrected as suggested*

Page 3: in the same paragraph as above, you may want to elaborate a little bit more on virus species and genetic diversity, such as which viruses have been proven to be transmitted and which have not been proven to be transmitted by the SSA1 whiteflies and their efficiency.

**Response:**

*Done as suggested. Current knowledge shows that all the cassava mosaic begomoviruses (CMBs) in Africa are transmitted by SSA1 and studies are on-going to study the efficiency of other SSA species* *.*

Page 3: Should be consistent in using P-endosymbiont, rather than P-symbiont and in other locations in the same document using P-endosymbiont.

**Response:**

*P-endosymbiont has been added throughout the text.*

Page 3: You stated “Seven facultative S-endosymbionts,” but only six endosymbionts are actually listed, need to add the missing one.

**Response:**

*Orientia-like organism added as the seventh endosymbionts*

Page 3: In the sentence starting with “It is against this background that we…” Your goal was to study whitefly-virus interaction, it is rather surprising that not a single virus sequence read was detected in these RNA-seq datasets in field-collected whiteflies. Rather than speculating these individual whitefly did not carry the target viruses, why not doing some tests by RT-PCR to confirm the lack of target viruses in these RNA preparations?

**Response:**

*Due to very little RNA (~17 uL) all was used in the cDNA library preparation; thus no further experiments could be done after library preparation. Additionally, the fact that we were analysing a single whitefly, the very low viral titre may not have been detectable. It is also known that RNA viruses such as CBSVs are picked up and kept for short periods in the whitefly stylet, unlike the DNA viruses that build-up and keep long in the midgut, and are more likely to be detected if present in the whitefly under study. Studies have shown that less than 10% of field whiteflies are viruliferous in any sample, and therefore it is possible that we missed the infected individuals during sampling*. *The suggestion would be to use freshly-collected samples in the future.*

IN METHODS:

Page 4: Since whiteflies were collected from CBSD-symptomatic cassava plants in Uganda and Tanzania, it might still be possible to conduct RT-PCR tests to determine the presence of viruses in the purified whitefly RNA preparations.

**Response:**

*It may not be possible to detect CBSVs even with RT-PCR because of the reasons provided above.*

Page 4: The rationale in using the Brazilian whitefly sample? Also it appears to me that there were no analysis of sequences from this Brazilian dataset in the result section.

**Response:**

*We have added further information regarding the Brazilian whitefly to the end of the first paragraph in the methods section.*

IN RESULTS:

Page 5: There were 65,550 to 162,487 contigs generated from individual RNA-seq datasets. How all these sequences could be assigned to? Any ideas on what proportion of the sequences belonging to whitefly *B. tabaci *genome, what proportion of the sequences to endosymbionts?

**Response:**

*We have added an additional line to Table 1 to show the number of endosymbiont contigs- thank you for this suggestion.*

Page 5: The citation to Table 2 seems to point to the listing of five endosymbionts, however the content in the Table 2 showing the origin of whiteflies collected.

**Response:**

*Corrected Table 2 with the prevalence of the endosymbionts included.*

Page 5: What are these incidences (74.8%, 71.2%, 54.1% and 58.5%) mean? The meaning is not clear, a proportion of total endosymbiont sequences that assigned to *P. aleyrodidarum*?

**Response:**

*For clarity, the absolute counts of the contigs associated with each endosymbiont have been provided rather than percentages and are linked directly to Table 2.*

Page 5:  The (data not shown) is not a good idea, it would be good to present it as a supplementary file.

**Response:**

*The mtCOI sequences were extracted from the transcriptomic data and then whiteflybase.org was used to verify the species ID.*

IN DISCUSSION:

Page 8: As mentioned in the general comment, you developed a method to effectively conduct RNA-sequencing on little RNA from individual whitefly and sequence analysis on specific genes in endosymbionts and COI gene of whitefly, I wouldn’t over use the term transcriptome analysis.

**Response:**

*RNA sequencing has been used instead of transcriptome sequencing where possible.*

Page 8: The deletion of 11 amino acids on the *Nus *G gene, this could be resulted from sequence assembly from short reads, therefore a validation of the deletion sequence on these whiteflies by RT-PCR and Sanger sequencing would be necessary, as pointed out by the authors that field collected samples may have resulted in RNA degradation in RNA purification, cDNA library preparation and/or sequencing.

**Response:**

*Previous studies and Genbank records (CP004358, LN649236, LN649255, LN734649) – from independent studies- have proven this is a legitimate deletion. Therefore, confirmation via further experiments is not needed in our opinion.*

Page 9: The sentence starts: “In addition, 51 and 21 reported…”, what do these numbers (51 and 21) mean here? If there are citations to references, you need to use author name followed by the reference number.

**Response:**

*Rephrased to read “In addition Arsenophonus has been reported within SSA1 B. tabaci in Eastern Africa collected from cassava* *[20, 50]* *.”*

Page 9: Tomato leaf curl virus should be changed to tomato yellow leaf curl virus. 

**Response:**

*Corrected as suggested.*

IN CONCLUSION:

Page 9: Suggest changing transcriptome sequencing to RNA sequencing.

**Response:**

*Done*

Page 9: In this study, you only worked with whiteflies, in the sentence you need to change “is” to “could be optimized and applicable to …”

**Response:**

*Rephrased as requested* *.*

Page 9: When you mentioned it is useful to study vector-microbiota-viral dynamics, but why not a single viral sequence read was detected in these datasets? Although there are some advantage in using RNA sequencing of individual whitefly, you may also want to point out there are still some room for improvement. 

**Response:**

*A sentence has been added on future improvements “* *However this method still requires further optimisation to recover viral reads especially in cases with very low viral titre as observed in this study”.*
